# Adherence to Guideline Recommendations in Patients with Thyroid Nodules

**Published:** 2017-02-15

**Authors:** Rudruidee Karnchanasorn, Kristine Grdinovac, Nichole Smith, Bhairvi Jani, G. John Chen

**Affiliations:** 1 University of Kansas Medical Center, Department of Internal Medicine, Division of Metabolism, Endocrinology and Genetics, Kansas City, KS; 2 Medical University of South Carolina, Division of Endocrinology, Diabetes & Medical Genetics, Charleston, SC; 3 Saint Louis University, Division of Gastroenterology and Hepatology, Saint Louis, MO; 4 University of Kansas Medical Center, Department of Internal Medicine, Division of Health Services Research, Kansas City, KS

**Keywords:** thyroid nodules, ultrasound-guided fine needle aspiration, thyroid stimulating hormone

## Abstract

**Introduction:**

Thyroid nodules are common and fine-needle aspiration (FNA) biopsy is the standard of care for work-up to exclude thyroid cancer. In this study, we examined the discrepancy between daily practice and recommended diagnostic approach for management of thyroid nodules, based on history taking, laboratory, and imaging studies.

**Methods:**

This was a retrospective chart review of 199 patients who had ultrasound-guided fine needle aspiration (UGFNA) performed at a Midwest academic medical center from January 2010 to December 2011. The quality measures were selected based on recommended clinical practice guidelines, including family history, history of neck radiation, neck symptoms, TSH test, and thyroid ultrasound.

**Results:**

The majority of patients were Caucasian females. Family history of thyroid cancer and childhood neck radiation exposure were documented in 79 subjects (40%) and 76 subjects (38%), respectively. Neck symptoms were documented in most subjects, including dysphonia (56.8%), dysphagia (69.9%), and dyspnea (41.2%). Most subjects had a TSH measured and an ultrasound performed prior to biopsy (75% and 86%, respectively).

**Conclusions:**

It appears there is a gap between current patient care and clinical practice guidelines for management of thyroid nodules. Clinical history and ultrasound features for risk stratification of UGFNA were lacking, which could reflect physicians’ unfamiliarity with the guidelines. As thyroid nodules are common, enhancing knowledge of the current guidelines could improve appropriate work-up. Further studies are needed to identify factors associated with the poor compliance with clinical guidelines in management of thyroid nodules.

## Introduction

Thyroid nodules are very common with an estimated prevalence of 4 – 7% by palpation^1^–^3^ and up to 30 – 60% based on autopsy studies.^3^ Fine-needle aspiration (FNA) biopsy is the standard of care for work-up and exclusion of thyroid cancer. Around 5 – 15% of thyroid nodules are malignant, but the malignancy risk increases based on age, sex, history of head and neck radiation, and family history.^1^,^4^ The incidence of thyroid cancer increased by 2.4 fold from 1973 to 2002^5^, which is likely due to increasing use of ultrasound (US). Various practice guidelines for management of thyroid nodules have been published, including the most recent 2015 American Thyroid Association (ATA)^6^ guideline. Clinical risk factors of malignancy include childhood radiation exposure, rapid growth of a nodule, hoarseness, vocal cord paralysis, dysphagia, or a family history of thyroid cancer or multiple endocrine neoplasia syndromes. Suspicious US features include hypoechogenicity, microcalcifications, taller than wide on transverse view, or infiltration of the surrounding tissue. ATA recommends FNA based on sizes and sonographic patterns, starting at 1 cm with intermediate to high suspicion patterns.

A serum thyroid-stimulating hormone (TSH) level should be obtained in all patients. If TSH is suppressed, a radionuclide thyroid scan (I-SCAN) should be obtained. FNA generally is not indicated for hyperfunctioning nodules due to low likelihood of malignancy.

In this study, we examined the discrepancy between daily practice and recommended diagnostic approach to thyroid nodules, based on history taking, laboratory, and imaging studies.

## Methods

Patients who underwent ultrasound-guided FNA (UGFNA) at our institution during January 1, 2010 to December 31, 2011 were retrospectively reviewed. The date range was chosen to begin following the revised ATA guideline published in 2009. We randomly selected 200 subjects and one patient was excluded due to duplicate records. Patients with previous history of thyroid cancer or thyroid surgery were excluded. The study protocol was approved by the local institutional review board. Data abstraction from the electronic medical record were entered in REDcap^7^, including demographic information, relevant clinical history including family history of thyroid cancer, history of childhood neck radiation, neck symptoms, TSH values, suspicious ultrasound characteristics, and I-SCAN results. The quality measures were determined based on five categories: (1) documentation of family history of thyroid cancer, (2) documentation of history of childhood neck radiation, (3) documentation of neck symptoms including dysphonia, dysphagia, and dyspnea, (4) presence of TSH values prior to UGFNA, and (5) presence of thyroid US prior to UGFNA.

The ultrasound results were categorized according to nodules sizes, number of nodules, echogenicity, presence of microcalcification, presence of irregular margins, presence of suspicious cervical lymph nodes, and evidence of growth from previous US. All analyses were conducted using SAS statistical software, version 9.4 (SAS Institute, Inc., Cary, NC, USA).

## Results

There were 833 UGFNA cases performed at our institution during January 1, 2010 to December 31, 2011. The 199 randomly-selected patients represented 273 FNA cases. The study group consisted of 37 (18.6%) males and 162 (81.4%) females, with a mean age of 55 ± 14 years. Of these, 72.9% were Caucasian. Forty-four percent had hypertension, 18.6 % had type 2 diabetes mellitus, and 13.6% had coronary artery disease.

Nearly 40% were questioned about family history of thyroid cancer and childhood neck radiation history. Neck symptoms were assessed for dysphonia, dysphagia, and dyspnea in 58%, 70%, and 39% of subjects, respectively (Table 1). TSH value was documented in 75%; 86% of patients had an ultrasound performed prior to biopsy. The ultrasound descriptions of 273 thyroid nodules are summarized in Table 2. None specified nodules which were taller than wide on transverse view. I-SCAN was performed in 10% of subjects.

Most FNA specimens were categorized as benign (81%). Indeterminate cytology, cytology suspicious for malignancy, and malignant cytology were found in 8%, 6%, and 1% of subjects, respectively. Surgery was performed on 78 subjects (29%), and 14 subjects (7%) were confirmed to have thyroid cancer.

## Discussion

The results showed that history-taking to identify clinical risk factors for thyroid cancer were lacking in more than 50% of patients. The majority of patients appropriately had TSH value and thyroid ultrasound performed prior to UGFNA as recommended by the guidelines. There was also significant discrepancy among sonographic description of thyroid nodules.

Tangpricha et al.^8^ reported in 1999 that the American Association of Clinical Endocrinologists guidelines had not been implemented fully among patients that presented with thyroid nodules. They also found that endocrine referral increased the rate of FNA performance, and the use of I-SCAN seemed to be over utilized (90%).^8^ The majority of our subjects were evaluated by primary care physicians and otolaryngologists, not endocrinologists. I-SCAN was performed less frequently in our study, which reflected a minority of patients with low TSH. Radionuclide studies have become less utilized at present time, most likely due to the evolution of thyroid ultrasound and increased availability of FNA.^9^

The retrospective chart review design limits the interpretation of the findings. Our study is limited to FNA cases from our institution and could underestimate the number of biopsies performed at an outside institution. In this current study, we examined the discrepancy between daily practice and recommended guidelines. In future studies, we plan to examine whether adherence to guidelines could impact early discovery of thyroid cancer.

A gap was revealed between current patient care and the recommended approach for management of thyroid nodules. Clinical history and US features for risk stratification of UGFNA were lacking, which could reflect physicians’ unfamiliarity with clinical practice guidelines. As thyroid nodules are common, improvement of knowledge of the current guidelines would be beneficial.

## Figures and Tables

**Table 1:** Findings of concordance quality measures.

Concordance Quality Measures	Documented (%)
Family History of Thyroid Cancer	79 (39.7)
Childhood Radiation Exposure	76 (38.2)
Symptoms of Dysphonia	113 (56.8)
Symptoms of Dysphagia	139 (69.9)
Symptoms of Dyspnea	82 (41.2)
Presence of TSH values prior to UGFNA	149 (74.9)
Presence of thyroid US prior to UGFNA	171 (85.9)

**Table 2:** Findings of ultrasound description of thyroid nodules.

Ultrasound Description	Number of Nodules (%)
Consistency (presence of solid, cystic, or mixed nodules)	135 (49.6)
Echogenicity	98 (36.0)
Presence or Absence of Microcalcification	52 (19.1)
Margin of Nodules	112 (41.1)
Cervical Lymphadenopathy	146 (53.7)
Evidence of Growth	80 (29.4)
